# Spermidine Increases the Sucrose Content in Inferior Grain of Wheat and Thereby Promotes Its Grain Filling

**DOI:** 10.3389/fpls.2019.01309

**Published:** 2019-11-21

**Authors:** Jian Luo, Bin Wei, Juan Han, Yuncheng Liao, Yang Liu

**Affiliations:** College of Agronomy, Northwest A&F University, Yangling, China

**Keywords:** polyamine, grain filling, wheat, carbohydrate transport, hormone

## Abstract

The improvement of grain filling is the key issue for promoting wheat thousand grain weight and grain yield. The levels of polyamines (PAs) significantly affect grain filling in cereals, but the mechanism by which PAs affect grain filling in wheat is unclear. In the present study, six wheat cultivars whose grain filling differed were used, and their grain-filling characteristics and endogenous PA contents were measured. In addition, exogenous PAs were supplied during the wheat grain-filling period. The grain-filling characteristics, hormone levels, starch contents, and gene expression [based on RNA sequencing (RNA-seq)] in the grain were analyzed. The objective of the present study was to investigate the effects of PAs on grain filling in wheat. The results suggested that the direct synthetic pathway from putrescine (Put) to spermidine (Spd) in the grain was a key factor in promoting grain filling and thousand grain weight in wheat. Spd through regulates the grain-filling rate of inferior grain during the early grain-filling period to affecting the grain filling and thousand grain weight of wheat. The promotive effect of Spd on the grain filling of inferior wheat grain was notably related to carbohydrate metabolism in that grain. Spd significantly increased the zeatin (Z) + zeatin riboside (ZR) contents but reduced the ethylene (ETH) evolution rate in the inferior grain. In addition, Spd significantly increased the sucrose synthase (SS) and acid invertase (AI) activities in the inferior grain. These effects of Spd led to increased sucrose content in the inferior grain. These reasons might explain why Spd significantly promoted the filling and weight of inferior wheat grain.

## Introduction

Wheat (*Triticum aestivum* L.) is an important global cereal crop species worldwide, including within China, and promoting wheat grain production is essential for food security in China. The grain yield of wheat can be divided into three components: the number of spikes per unit area, the number of spikelets per spike, and thousand grain weight (Yu, 2011). In China, high wheat grain yield relies mainly on high numbers of spikes per unit area (Cai et al., 2014). However, the high numbers of spikes may lead to severe problems, such as lodging, premature senescence, and increased damage from disease and insects (Sicher and Bunce, 1998; Robert., 2002; Kelbert et al., 2004). Therefore, increasing the thousand grain weight or grain number per spike based on a suitable panicle number per area is an inevitable approach to promote wheat grain yield. In cereals, grain filling determines the thousand grain weight, so improving grain filling is important for achieving increased wheat thousand grain weight and grain yield (Kato et al., 2007).

Wheat grain can be divided into two types: superior grain and inferior grain. Superior grain consists mainly of early flowering types, usually located on basal of the middle spikelets, which produce larger and heavier grains, while inferior grain consists mainly of late-flowering types, usually located on distal of the middle spikelets and the basal and the distal spikelets, poorly to produce grains (Jiang et al., 2003; Yang and Zhang, 2010). Compared with superior grain, inferior grain requires more energy for grain filling (Peng et al., 2011), and inferior grain is more sensitive to environmental factors, such as water, temperature, and fertilizer (Peng et al., 2013). Therefore, the weight of inferior grain is low and notably varies from year to year (Yang and Zhang, 2010). Previous studies suggested that variations in the grain weight of cereals such as rice (*Oryza sativa* L.) and wheat are caused mainly by inferior grain (Yang and Zhang, 2006). Thus, improving the filling of inferior grain is key for promoting wheat grain weight and grain yield.

Carbohydrates are the main components of wheat grain and account for more than 70% of grain dry weight (Yang et al., 2004). The carbohydrates that accumulate in wheat grain are derived mainly from the transportation of nonstructural carbohydrates (NSCs) stored in the stem (Sikder and Gupta, 1976). Previous studies have suggested that superior grain and inferior grain notably differed in their ability to use NSCs stored in the stem (Murty and Murty, 1982). Compared to those of superior grain, the lower sink size and sink strength of inferior grain limit the transport of NSCs from the stem; this phenomenon leads to the inferior grain being a poor sink for NSCs stored in the stem, which is one of the main reasons why in cereals, compared with superior grain, inferior grain experiences poor filling and weighs less (Fu et al., 2011; Liang et al., 2017). Appropriate nitrogen (N) and potassium (K) fertilizer applications significantly promoted NSC transport from the stem to inferior grain and notably increased the weight of inferior grain of wheat and rice (Fu et al., 2011; Yang and Zhang, 2010; Liang et al., 2017). This finding means that promoting NSC transport from the stem to inferior grain may be an effective way to improve the grain filling of inferior grain in cereals.

Polyamines (PAs) are important endogenous plant growth regulators and notably regulate the grain filling of cereals such as wheat, rice, and maize (Kusano et al., 2007; Yang et al., 2008; Liu et al., 2013). Previous studies have suggested that PA biosynthesis is significantly related to the grain-filling rate and grain weight in rice; high spermidine (Spd, a type of PA) and spermine (Spm, a type of PA) levels in grain were beneficial to the grain filling of inferior spikelets in rice (Yang et al., 2008; Chen et al., 2013). Severe water deficit significantly reduced Spd levels in grain and inhibited grain filling in wheat (Liu et al., 2016; Yang et al., 2017). External Spd and Spm promoted wheat grain filling (Liu et al., 2013). These findings indicated that PAs affect grain filling in cereals. However, the mechanism by which PAs affect grain filling in wheat is unclear.

Previous study has suggested that PAs are notably related to carbohydrate metabolism in plants (Oufir et al., 2008). Chen et al. (2013) suggested that PAs promote starch synthesis and consequently promote grain filling in rice. Liu et al. (2013) suggested that PAs increase both the photosynthetic rate in flag leaves and carbohydrate accumulation in plants, thereby promoting grain filling in wheat. However, whether the effect of PAs on the grain filling of wheat is related to NSC transport from the stem to the grain is also unclear. The previous study suggested that the superior grains of wheat showed generally higher activity of sucrose synthase (SS, EC 2.4.1.13) than inferior grains, and this may relate to the higher starch accumulation rates and grain weight of superior grain (Jiang et al., 2003). Wang et al. (2017) suggested that sucrose-phosphate synthase (SPS, EC 2.4.1.14) activity in straws was important for carbon reserve remobilization of rice. Beside this, the acid invertase (AI, EC 3.2.1.26) was related to the sucrose unloading in grain, and the nitrogen application notably promoted the AI activity in wheat grain and increased the sugar content in grains (Zhang et al., 2014). These mean that these enzymes were notably involved in the sugar transport of cereals. But, whether the PA through regulated the activities of these enzymes, such as SS, SPS and AI, to affect the grain filling of wheat, is also unclear. In addition to carbohydrates, hormones, plant senescence, and N metabolism are also considered to regulate grain filling in cereals Takahashi et al., 1996; Kim et al., 2011;Wilkinson et al., 2012). Previous studies have suggested that the effect of PAs on the grain filling of cereals was related to hormone and plant senescence (Yang et al., 2008), which means that there may be an interaction among PAs, hormones, carbohydrate metabolism, and plant senescence in the regulation of wheat grain filling. However, little is known about the regulatory network of PAs on the grain filling of inferior wheat grain. RNA sequencing (RNA-seq) has become an essential method for large-scale analysis of genes in various fields of plant biology, including grain filling in cereal crops. Li et al. (2014) identified 7713 differentially expressed genes (DEGs) in grain-filling caryopses between the rice *sugary* mutant and the wild-type strain based on RNA-seq data. Moreover, using RNA-seq, Jeong et al. (2017) studied phosphorus (P) remobilization from rice flag leaves during grain filling, and Peng et al. (2011) suggested that the expression and function of miRNA partly explain the slow grain-filling rate of inferior spikelets. However, little is known about the mechanism of grain filling in wheat *via* RNA-seq analysis.

In the present study, six wheat cultivars whose grain filling differed were used, and the grain-filling characteristics, endogenous PA content, and the activities of the enzymes that regulated PA synthesis were measured. In addition, exogenous Spd and putrescine (Put) were supplied during the wheat grain-filling period. The objective of the present study was to investigate the effect of PAs on the grain filling of inferior grain in wheat. For this, the grain-filling characteristics of the grain and gene expression (based on RNA-seq) were analyzed, and the endogenous zeatin (Z) and zeatin riboside (ZR) contents, the ethylene (ETH) evolution rate, the starch and sucrose contents, the activities of the enzymes involved in sucrose–starch synthesis, and carbohydrate transport from stem to grain were measured.

## Materials and Methods

### Experimental Design

Two experiments were conducted at the experimental site of Northwest A&F University, Yangling, Shaanxi Province, China (34°17’ N, 108°05’ E) in 2014–2016, and the two experiments were conducted in the same experimental field. The soil at the experimental site is an Eum-Orthrosol (Chinese soil taxonomy). The organic matter content and available N, P, and K in the 0–20 cm of topsoil in the cropland were 12.39 g kg^−1^, 49.85 mg kg^−1^, 24.63 mg kg^−1^, and 110.14 mg kg^−1^, respectively.

The First Experiment

Six winter wheat cultivars, Shuangda 1 (SD 1), Fugao 1 (FG 1), Zhoumai 22 (ZM 22), Xiaoyan 6 (XY 6), Xiaoyan 22 (XY 22), and Xinong 538 (XN 538), were sown at a rate of 150 kg ha^−1^, and the row spacing was 0.25 m. The sow date was October 16 in 2014–2015 and 2015–2016. Urea and (NH_4_)_2_HPO_4_ were applied and each of them applied at 375 kg ha^−1^. All fertilizer applied was at a basal level. The experiment was conducted in accordance with a completely randomized design, and each treatment consisted of three replicates. The area of each plot was 6 m^2^ (3 × 2 m).

The Second Experiment

Two wheat cultivars, SD 1 and XN 538, were sown. The planting method was the same as that of the first experiment. At anthesis, four treatments were applied to each cultivar: SPD, the spikes were sprayed with 1 mmol L^−1^ Spd at anthesis; Put, the spikes were sprayed with 2 mmol L^−1^ Put at anthesis; MGBG (Yang et al., 2008), the spikes were sprayed with 5 mmol L^−1^ methylglyoxal-bis(guanylhydrazone) (MGBG, an inhibitor of Spd) at anthesis; and CK, the spikes were sprayed with water at anthesis. Spd, Put, MGBG, and water were applied daily for four days at a rate of 5 ml spike^−1^ for each application. All of the solutions contained 0.1% (V/V) ethanol and 0.01% (V/V) Tween-20. For the CK, the water contained the same concentrations of ethanol and Tween-20. Each treatment was replicated three times in a split-plot experimental design. The area of each plot was 5 m^2^ (2.5 × 2 m). Two cultivars, four chemical application treatments, and three replicates gave 24 individual plots. The Spm, Spd, and MGBG were purchased from Sigma-Aldrich (USA).

### Sampling and Measurements

For each treatment, the spikes that flowered on the same day were tagged and sampled from anthesis to maturity at 4-day intervals. Forty spikes were sampled at each sampling stage for each plot.

The grain on an ear was divided into superior grain and inferior grain according to the methods of Jiang et al. (2003). Half of the sampled grain was quickly frozen in liquid N and then stored at −80°C, and the enzymes within the other half were deactivated by heating at 105°C for 30 min, after which the grain was then dried at 70°C to a constant weight and subsequently weighed. The yield and yield components were determined according to our previous methods (Liu et al., 2016).

#### Grain-Filling Process

The grain-filling process was fitted by Richards’ (1959) growth equation as described by Zhu et al. (1988).

(1) $$W=\frac{A}{{\left(1+Be^{kt}\right)}^{}}$$

The grain ﬁlling rate (*G*) was calculated using a derivation of Equation 1:

(2) $$G=\frac{AkBe^{-kt}}{{\left(1+Be^{-kt}\right)}^{\left(\frac{N+1}{N}\right)}}$$

where *W* is the grain weight (mg); *A* is the ﬁnal grain weight (mg); *t* is the time after anthesis (d); and *B*, *k*, and *N* are coefficients determined using regression.

The active grain filling period was defined as the period when *W* was between 5% (*t*_1_) and 95% (*t*_2_) of A. Therefore, the average grain filling rate during this period was calculated from *t*_1_ to *t*_2_ (Yang et al., 2006).

#### PAs

Spd, Spm, and Put were extracted and measured according to the methods of Liu et al. (2002). Brieﬂy, approximately 0.5 g fresh weight (FW) of samples was homogenized in 3–5 ml of 5% (v/v) perchloric acid (PCA) in a prechilled mortar and pestle. The Spd, Spm, and Put were measured according to the methods Liu et al. (2016) and quantiﬁed *via* a high-performance liquid chromatography system (Waters 1525 Binary HPLC Pump/2489 UV Detector, Waters, USA).

#### PA Biosynthetic Enzyme Activity

The activities of arginine decarboxylase (ADC, EC 4.1.1.19), S-adenosylmethionine decarboxylase (SAMDC, EC 4.1.1.50), and Spd synthase (SpdSy, EC 2.5.1.16) were measured according to the methods of Yang et al. (2008). The activities of ADC and SAMDC were determined by measuring the CO_2_ evolution as described by Lee et al. (1997), and the SpdSy activity was assayed according to the methods of Kasukabe et al. (2004).

#### RNA Extraction, Library Preparation, and RNA-Seq

At 8 and at 16 days post-anthesis, four tagged spikes in each plot were used, respectively. The inferior grain of these spikes was sampled, after which it (the inferior grain of 8 and 16 days post-anthesis) was mixed together and then used for RNA-seq analysis. The RNA extraction, library preparation, and RNA-seq were performed by Novel Bioinformatics Ltd., Co. (Shanghai, China). The total RNA was isolated using TRIzol reagent (Invitrogen, Carlsbad, CA), and the RNA quality was confirmed using a microspectrophotometer (NanoDrop™ 2000, Thermo Fisher Scientific, MA, USA).

A sequencing library of each RNA sample was prepared by using an Illumina TruSeq RNA Library Prep Kit v2 according to the protocol provided by the manufacturer (Illumina, USA). FAST-QC was then used to evaluate the quality of the sequencing data (Langmead et al., 2009; Trapnell et al., 2009). Raw reads after quality control testing were subsequently mapped to the reference wheat genome using the HISAT2 algorithm with the default parameters (Pertea et al., 2016).

A differential expression analysis was performed in which the EBSeq algorithm was used to filter DEGs by a log-fold expression change (log2 fold change (FC) > 0.585 or <−0.585) with a false discovery rate (FDR) threshold of <0.05. The DEGs were queried *via* BLAST using the tools and databases for the *T. aestivum* L. IWGSC1+popseq.31 genome assembly hosted on the EnsemblPlants website (http://plants.ensembl.org/Triticum_aestivum/Info/Index). Gene Ontology (GO) analysis was applied to analyze the main functions of the DEGs according to the GO database, which provides key functional classifications for genes (Ashburner et al., 2000). Pathway assignments were carried out based on the Kyoto Encyclopedia of Genomes and Genomes (KEGG) database (www.genome.ad.jp/kegg/). The RNA-seq analysis had three biological replicates, and the RNA-seq data were deposited in the Sequence Read Archive of NCBI; the accession number is SRP217735.

#### Sucrose and Starch Contents and the Activities of Enzymes Involved in Starch Biosynthesis in the Grain

Sucrose was extracted from the grain by 80% ethyl alcohol and measured by the resorcinol-HCl method (Wang and Huang, 2000). After sucrose was extracted, the residue was extracted by 36 mol L^−1^ PCA, and then extracted by 18 mol L^−1^ PCA, and the extracting solutions were mixed and used for starch measured. The starch concentration was measured *via* the anthrone method (Liu et al., 2011).

The activities of SS, SPS, and AI in the grain or stems were measured according to the methods of Jiang et al. (2003) and Yang et al. (2004).

#### Hormones

Endogenous Z+ZR and abscisic acid (ABA) were extracted according to previous reports (Liu et al., 2011), using 80% (v/v) methanol. Z+ZR and ABA were quantified *via* by enzyme-linked immunosorbent assays (ELISAs) (Liu et al., 2011). The recovery rates for Z+ZR and ABA were 94.8 ± 5.7% and 92.4± 8.8%, respectively.

The ETH generated by the grains was determined according to the methods of Beltrano et al. (1994) and Yang et al. (2008). The ETH was assayed using a gas chromatography (GC) system (Trace GC UItra^™^, Thermo Fisher Scientific, USA) according to our previous study (Liu et al., 2016).

### Statistical Analyses

The SPSS 16.0 statistical software package was used to conduct ANOVAs. The data from each sampling were analyzed separately. The analysis used completely randomized design and split-plot experimental design, respectively, for experiments 1 and 2. The means were tested by the Tukey HSD test.

## Results

### Grain Filling

**Figure 1:**
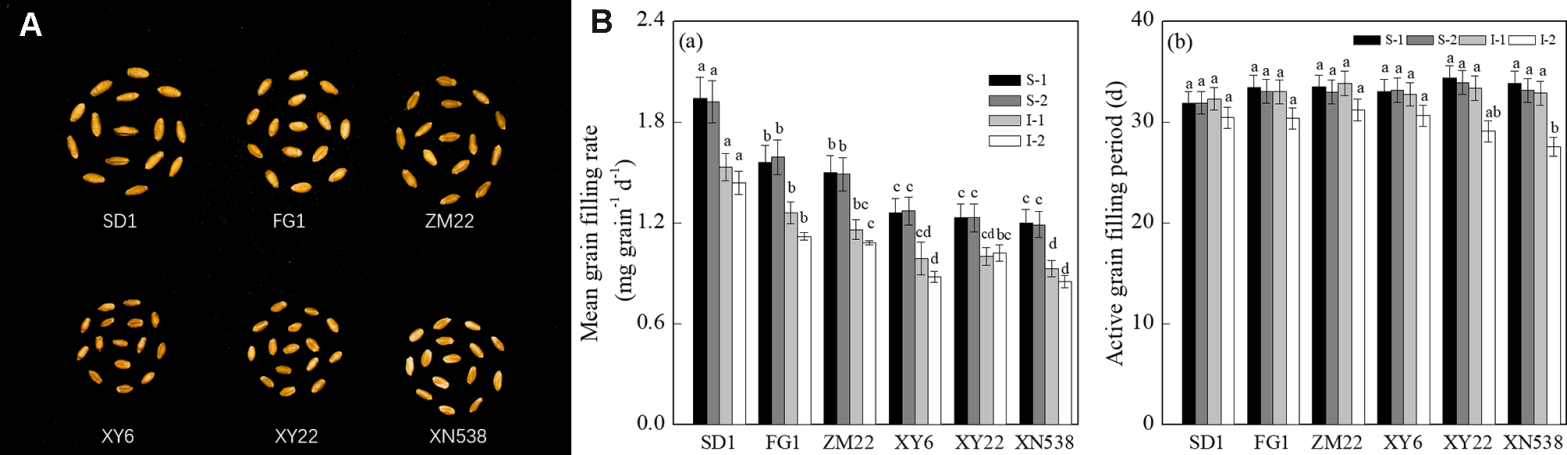
Grain-filling characteristics of different wheat cultivars. **(A)** The mature grains of the six wheat cultivars. **(B)** The mean grain-filling rate (a) and active grain-filling period (b) of the six wheat cultivars. Vertical bars represent ± the standard error of the mean (n = 3, n represents the biological replicates). Values for the same year and same grain type followed by different letters are significantly different (P 0.05). * represents the significant difference (P 0.05) between superior grain and inferior grain for the same year. SD 1, FG 1, ZM 22, XY 6, XY 22, and XN 538 are the cultivars Shuangda 1, Fugao 1, Zhengmai 22, Xiaoyan 6, Xiaoyan 22, and Xinong 538, respectively. S, superior grain; I, inferior grain; 1 and 2 represent 2014–2015 and 2015–2016, respectively.

**Figure 2:**
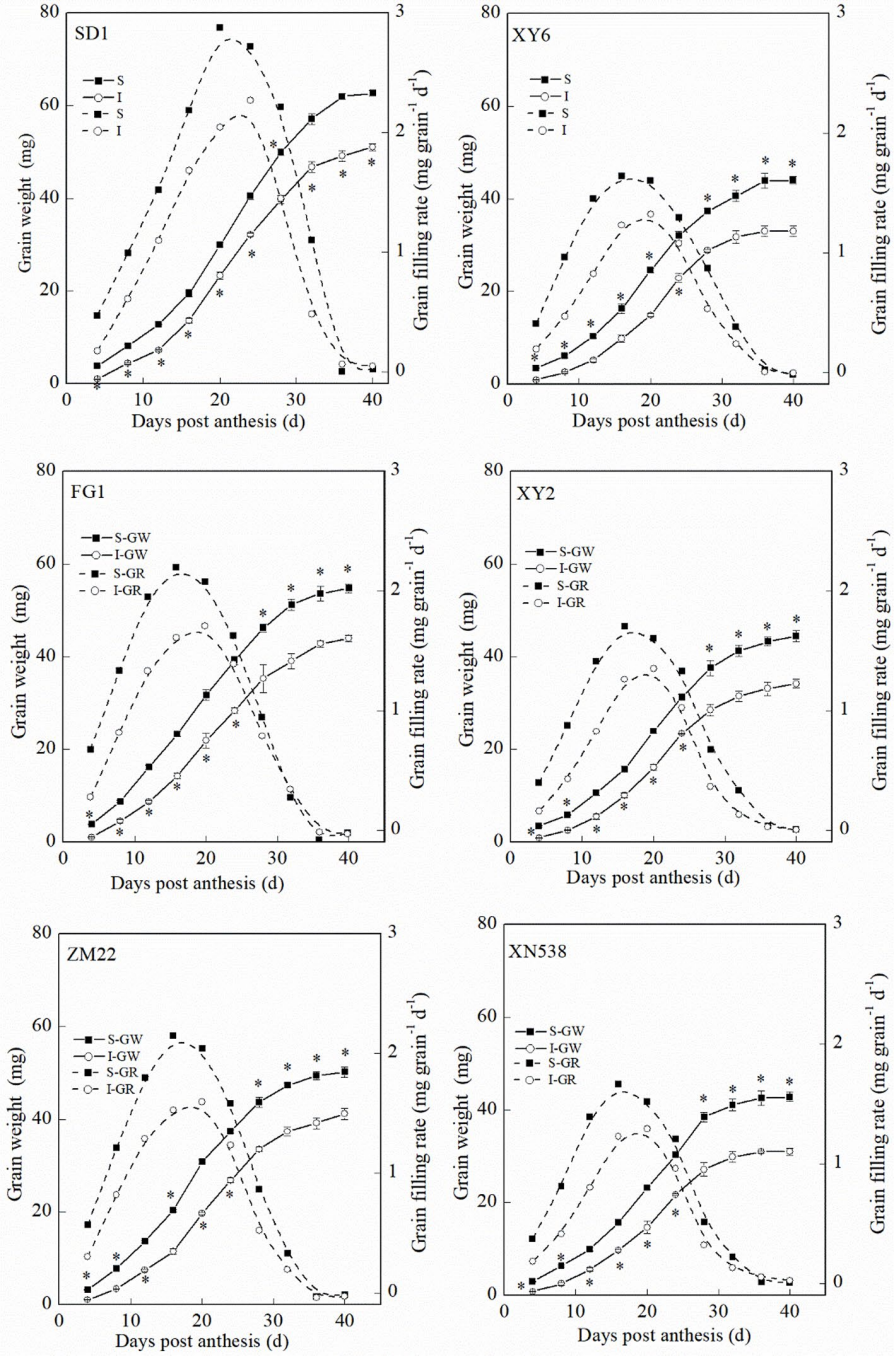
The changes of grain weight and grain-filling rate characteristics during grain-filling period. Vertical bars represent ± the standard error of the mean (n = 3, n represents the biological replicates). * represents the significant difference (P 0.05) between superior grain and inferior grain at the same day. The solid line and dashed line represent the grain weight and grain-filling rate, respectively. SD 1, FG 1, ZM 22, XY 6, XY 22, and XN 538 are the cultivars Shuangda 1, Fugao 1, Zhengmai 22, Xiaoyan 6, Xiaoyan 22, and Xinong 538, respectively. S, superior grain; I, inferior grain.

**Figure 1** and **Supplemental Table 1** show that the thousand grain weights of the six cultivars were significantly different. The mean grain-filling rate and thousand grain weight among the cultivars followed the order of SD 1 > FG 1 and ZM 22 > XY 6, XY 22, and XN 538 (**Figures 1A, B-a**). However, no significant differences in the active grain-filling period were observed between the six cultivars (**Figure 1B-b**). Among the six cultivars, the grain-filling rate and weight of the superior grain were significantly higher than those of the inferior grain of each cultivar (**Figure 2**). However, the trends of the active grain-filling period between the superior grain and inferior grain differed between the two years. In 2014–2015, no significant differences were observed for the active grain-filling period between the superior grain and inferior grain for any of the six cultivars. However, the active grain-filling period of the inferior grain was significantly lower than that of the superior grain in 2015–2016. These results suggested that compared with the superior grain, the significant low grain weight of inferior grain for these six cultivars seems due to the lower level of grain filling rate.

### PA Contents

**Figure 3:**
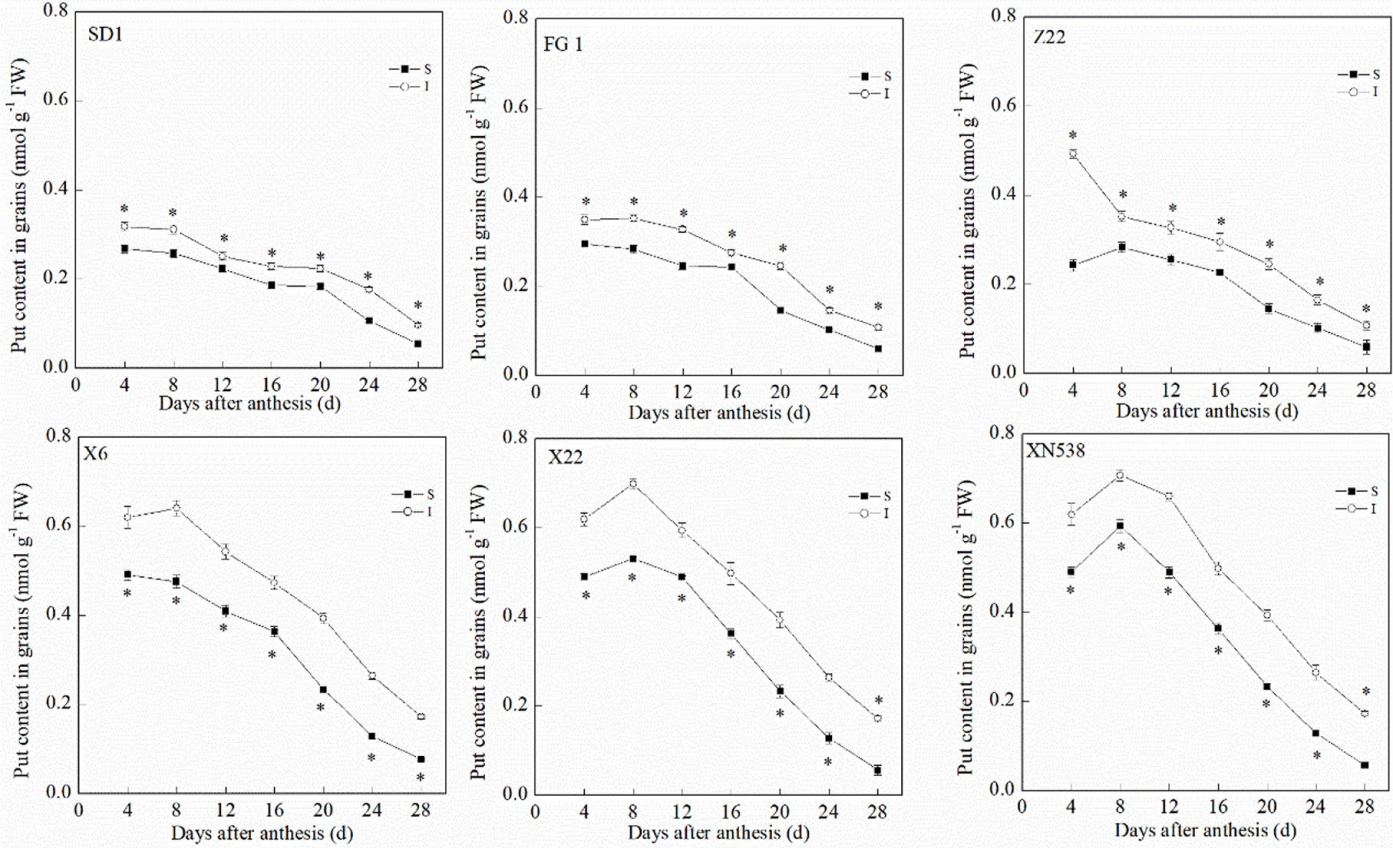
The changes of putrescine (Put) contents in wheat grains during grain-filling period. Vertical bars represent ± the standard error of the mean (n = 3, n represents the biological replicates). * represents the significant difference (P 0.05) between superior grain and inferior grain at the same day. SD 1, FG 1, ZM 22, XY 6, XY 22, and XN 538 are the cultivars Shuangda 1, Fugao 1, Zhengmai 22, Xiaoyan 6, Xiaoyan 22, and Xinong 538, respectively. S, superior grain; I, inferior grain.

**Figure 4:**
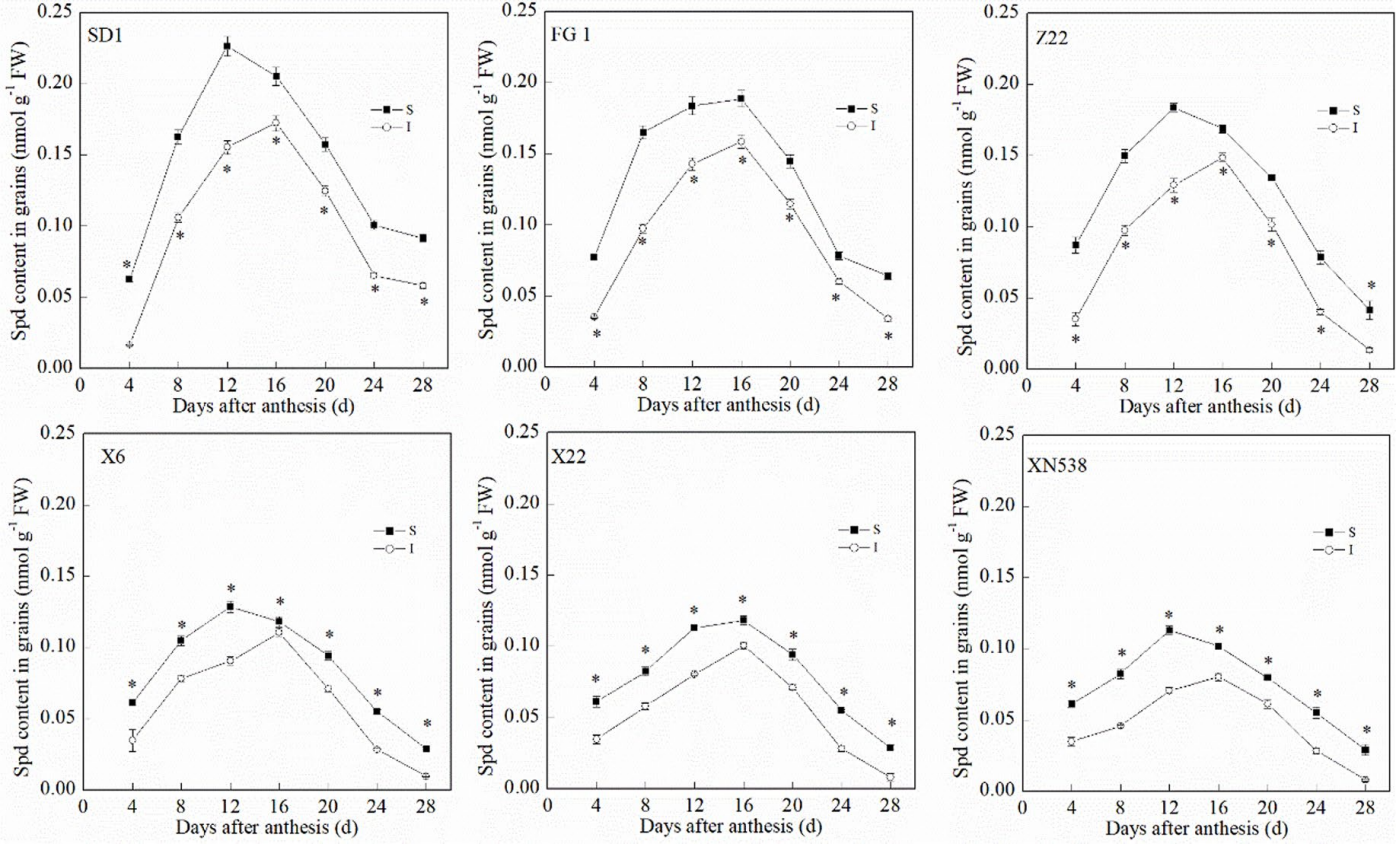
The changes of spermidine (Spd) contents in wheat grains during grain-filling period. Vertical bars represent ± the standard error of the mean (n = 3, n represents the biological replicates). * represents the significant difference (P 0.05) between superior grain and inferior grain at the same day. SD 1, FG 1, ZM 22, XY 6, XY 22, and XN 538 are the cultivars Shuangda 1, Fugao 1, Zhengmai 22, Xiaoyan 6, Xiaoyan 22, and Xinong 538, respectively. S, superior grain; I, inferior grain.

During the grain-filling period, the trends of the endogenous Put and Spd levels in the grain differed (**Figures 3** and **4**). The Put level in the grain decreased during grain filling (**Figure 3**). In addition, the Put level in the superior grain was notably lower than that in the inferior grain during the grain-filling period. With respect to the low-grain-weight cultivars, XY 6, XY 22, and XN 538, the Put levels in the grain were significantly greater than those of the high-grain-weight cultivars, SD 1, FG 1, and ZM 22, on the same day during the grain-filling period. In contrast to Put, the Spd in the grain first increased and then decreased during the grain-filling period, and the Spd peaked at 16 days post-anthesis (**Figure 4**). The Spd levels in the grain of the low-grain-weight cultivars, XY 6, XY 22, and XN 538, were significantly lower than those of high-grain-weight cultivars, SD 1, FG 1, and ZM 22, on the same day during the grain-filling period. The Spd levels in the superior grain were significantly greater than those in the inferior grain in all six cultivars. Correlation analysis revealed that the Spd levels in the grain were positively and significantly correlated with the grain-filling rate and thousand grain weight, but the Put levels in the grain were negatively and significantly correlated with the grain-filling rate and thousand grain weight (**Table 1**). However, the Put and Spd levels in the grain were not significantly correlated with the active grain-filling period.

**Table 1:** Correlation coefficients of peak polyamine (PA) contents in wheat grain for the two grain types (superior grain and inferior grain) and six cultivars with the mean grain-filling rate (MGR), active grain-filling period (AGP), and final grain weight (GW) of wheat.

	MGR	AGP	GW	Spd	Put
MGR	1				
AGP	0.5536	1			
GW	0.9865**	0.5680	1		
Spd	0.8838**	0.3661	0.8464**	1	
Put	−0.8283**	−0.4556	−0.8162**	−0.9486**	1

*Significant at the 0.05 probability level (n = 12). **Significant at the 0.01 probability level (n = 12). GW, the ﬁnal grain weight; MGR, mean grain-filling rates; AGP, active grain-filling period; Spd, spermidine; Put, putrescine. The n represents the treatment number; for the correlation analysis, there are six cultivars and two grain types, so it had 12 treatments.

The exogenous PAs affected the endogenous Pas’ concentration in grains (**Supplemental Table 2**). The exogenous Spd significantly increased the endogenous Spd concentration in inferior grain. Beside this, the exogenous MGBG significantly decreased the Spd concentration in superior grain and inferior grain.

### PA Biosynthetic Enzyme Activity

**Figure 5:**
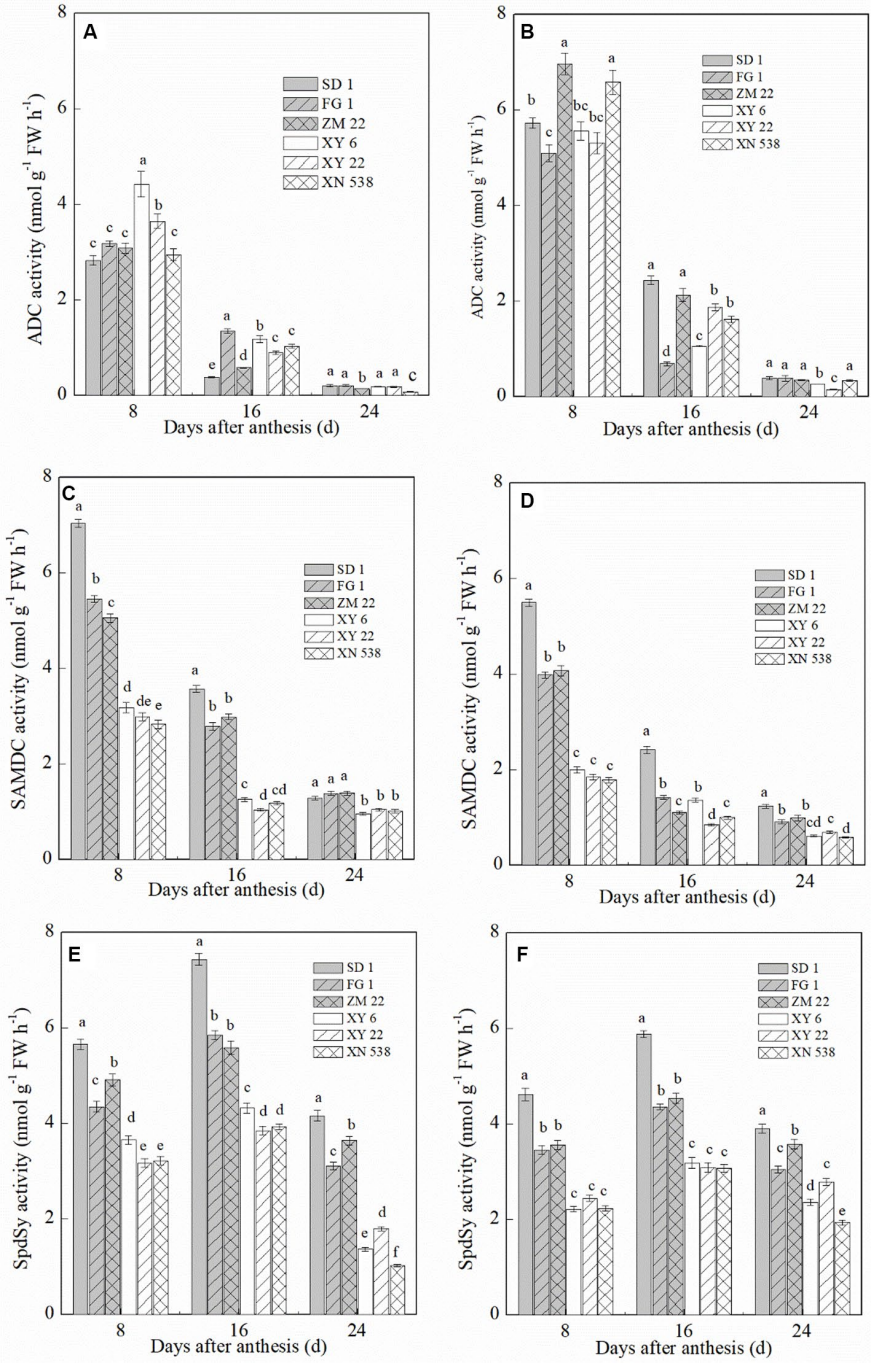
The changes of polyamine (PA) biosynthetic enzyme activity in wheat grains during grain filling period. Vertical bars represent ± the standard error of the mean (n = 3, n represents the biological replicates). Values for the same day followed by different letters are significantly different (P 0.05). SD 1, FG 1, ZM 22, XY 6, XY 22, and XN 538 are the cultivars Shuangda 1, Fugao 1, Zhengmai 22, Xiaoyan 6, Xiaoyan 22, and Xinong 538, respectively. **(A**, **C**, and **E)** represent the superior grain; **(B**, **D**, and **F)** represent the inferior grain.

The ADC, SAMDC, and SpdSy activities in the grain notably differed between the six cultivars (**Figure 5**). The ADC activity in the grain decreased during grain filling, and the ADC activity was significantly higher in the inferior grain than in the superior grain on the same day post-anthesis. In the superior grain, XY 6 exhibited the highest ADC activity at 8 days post-anthesis, but the highest ADC activity in the inferior grain on the same day occurred in ZM 22. In contrast to the ADC activity, the SAMDC and SpdSy activities exhibited a similar trend among the cultivars. Compared with the low-grain-weight cultivars, XY 6, XY 22, and XN 538, the high-grain-weight cultivars, SD 1, FG 1, and ZM 22, had higher SAMDC and SpdSy activities in their grain. In addition, the SAMDC and SpdSy activities were significantly lower in the inferior grain than in the superior grain on the same day post-anthesis.

## Effects of Exogenous Pas on Grain Filling

**Figure 6:**
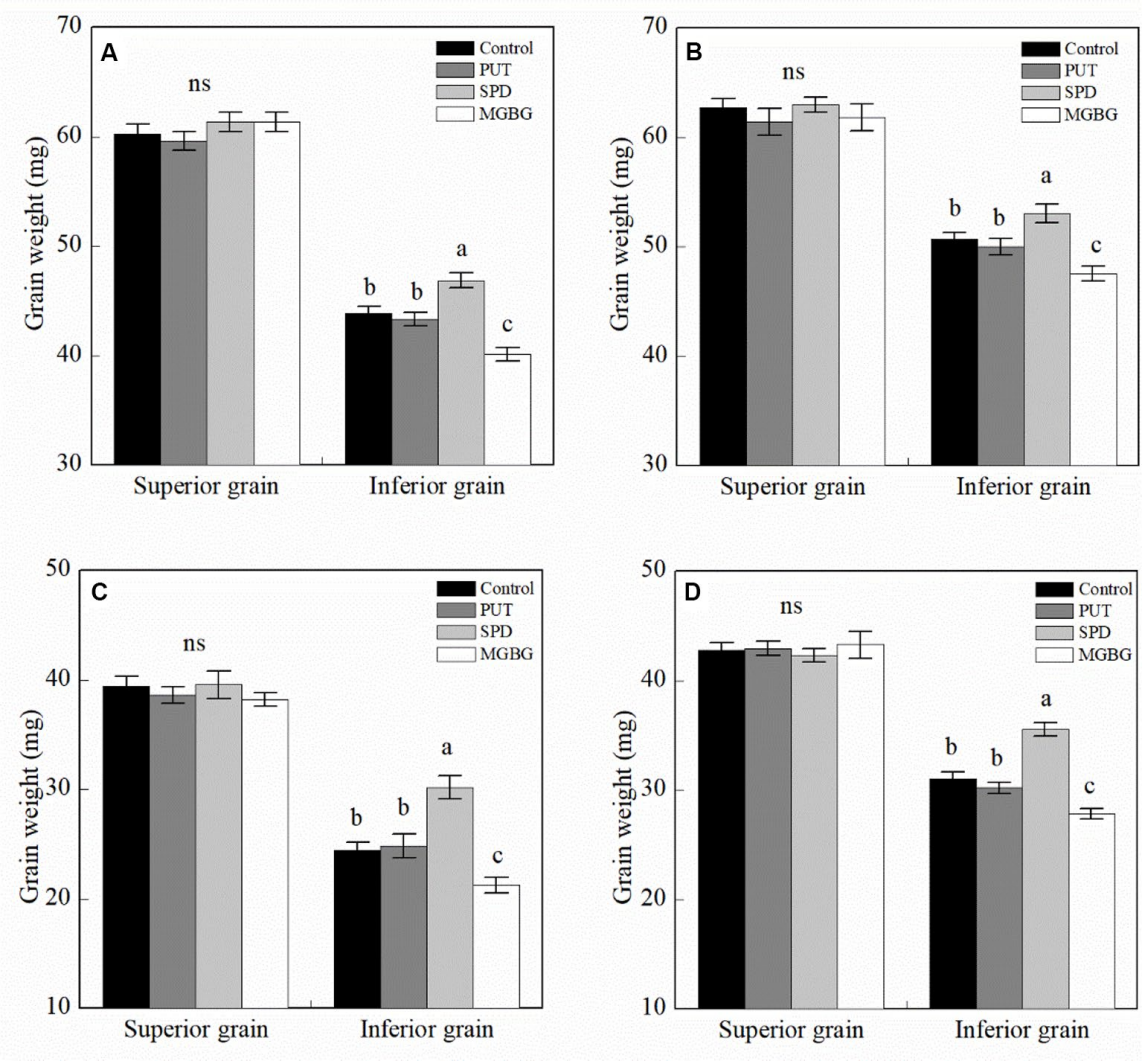
Effect of external PA on the grain weight of wheat. **(A** and **B)** Shuangda 1 at 2014–2015 and 2015–2016, respectively; **(C** and **D)** Xinong 538 at 2014–2015 and 2015–2016, respectively. PUT, SPD, MGBG, and control represent external applied Put, Spd, methylglyoxal-bis(guanylhydrazone) (MGBG), and water, respectively, to spikelets at anthesis stage. Vertical bars represent ± the standard error of the mean (n = 3, n represents the biological replicates). Values within the same grain type followed by different letters are significantly different (P 0.05); ns represents there being no significant difference among all of the treatments for the same grain type.

The results of 2-year experiments suggested that exogenous PAs significantly affected the thousand grain weight of the two cultivars (**Supplemental Table 3**). The exogenous PAs had no significant effect on the weight of superior wheat grain (**Figure 6**). However, exogenous Spd significantly promoted the increase in inferior thousand grain weight, and exogenous MGBG, an Spd synthesis inhibitor, significantly reduced the weight of the inferior grain. However, exogenous Put had no significant effect on the weight of the inferior grain.

**Figure 7:**
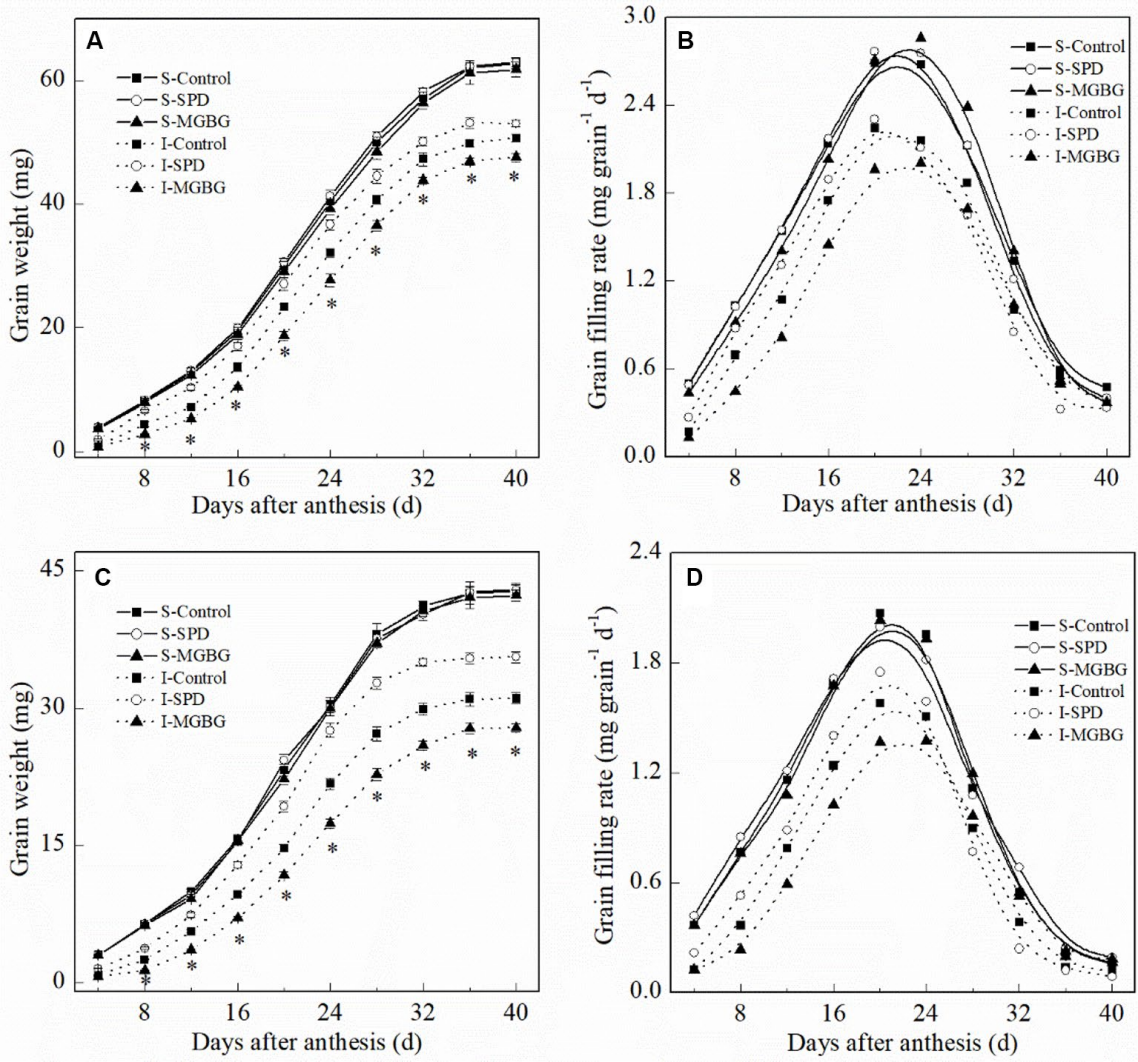
Effect of external PA on the changes of grain weight and grain-filling rate of Shuangda 1 **(A** and **B)** and Xinong 538 **(C** and **D)** during the grain-filling period. SPD, MGBG, and control represent external applied Spd, MGBG, and water, respectively, to spikelets at anthesis stage. Vertical bars represent ± the standard error of the mean (n = 3, n represents the biological replicates). * represents there being significant difference among the treatments for the same grain type (F value > F_0.05_). S, superior grain; I, inferior grain.

Exogenous Spd differentially affected the mean grain-filling rate and active grain-filling period (**Figure 7** and **Table 2**). Exogenous Spd and MGBG had no significant effect on the active grain-filling period of either the superior grain or inferior grain. However, exogenous Spd significantly promoted the mean grain-filling rate of the inferior grain, whereas exogenous MGBG had the opposite effect. The grain-filling rate of the inferior grain in the SPD treatment was greater than that in the CK treatment during 4–24 days post-anthesis.

**Table 2:** Effect of external PA on the grain-filling characteristics of wheat.

Cultivar	Spikelet categories	Treatment	MGR	AGP	GW
			mg grain^−1^ d^−1^	d	mg
Shuangda 1	S	CK	1.82a	35.94a	65.41a
		SPD	1.81a	35.85a	64.89a
		MGBG	1.80a	35.88a	64.58a
	I	CK	1.68b	31.27a	52.53b
		SPD	1.76a	31.56a	55.55a
		MGBG	1.56c	31.60a	49.30c
Xinong 538	S	CK	1.35a	32.20a	43.47a
		SPD	1.33a	33.04a	43.94a
		MGBG	1.33a	32.29a	42.95a
	I	CK	1.13b	27.93a	31.56b
		SPD	1.34a	26.84a	35.97a
		MGBG	0.96c	28.18a	27.05c

Values within a column and for the same cultivar and same grain type followed by different letters are significantly different (P < 0.05). GW, the ﬁnal grain weight; MGR, mean grain-filling rates; AGP, active grain-filling period; S, superior grain; I, inferior grain. SPD, MGBG, and CK represent external applied Spd, MGBG, and water, respectively, to spikelets at anthesis stage. The data list in the table is the average of the three biological replicates.

## Gene Expression of RNA-Seq Data

**Figure 8:**
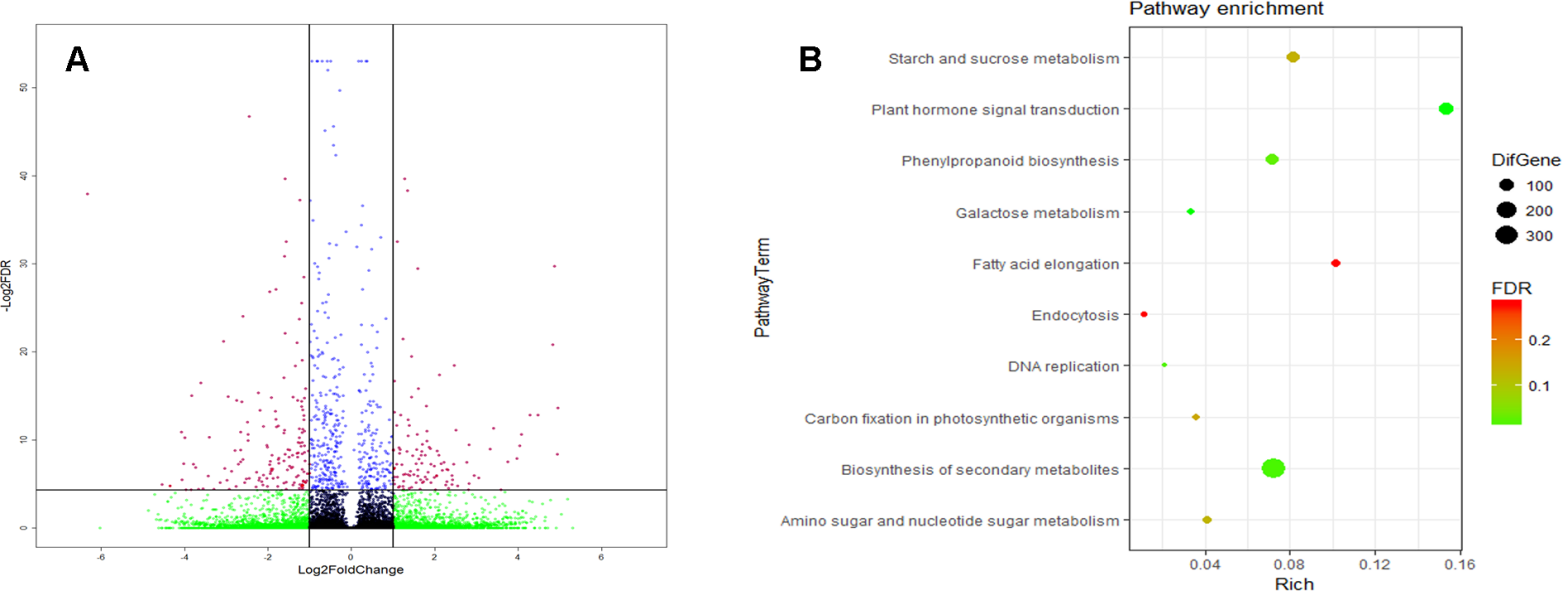
Gene expression base on the RNA sequencing (RNA-seq) analysis. **(A)** The red dot represents the differentially expressed genes (DEGs) between the SPD (spikes sprayed with 1 mmol L^-1^ Spd at anthesis and CK (spikes sprayed with water at anthesistreatment **(B)** The Kyoto Encyclipedia of Genomes and Genomes (KEGG) pathway of the DEGs [false discovery rate (FDR)<0.05]. The RNA-seq analysis had three biological replicates.

Compared to CK, the external Spd upregulated 167 genes’ expression, and it downregulated 282 genes’ expression (**Figure 8A**). KEGG analysis revealed that 317 DEGs were assigned to 10 KEGG pathways (FDR < 0.05) (**Figure 8B**). These KEGG pathways involve mainly starch and sucrose metabolism, plant hormone signal transduction, phenylpropanoid biosynthesis, secondary metabolite biosynthesis, fatty acid elongation, etc.

**Figure 9:**
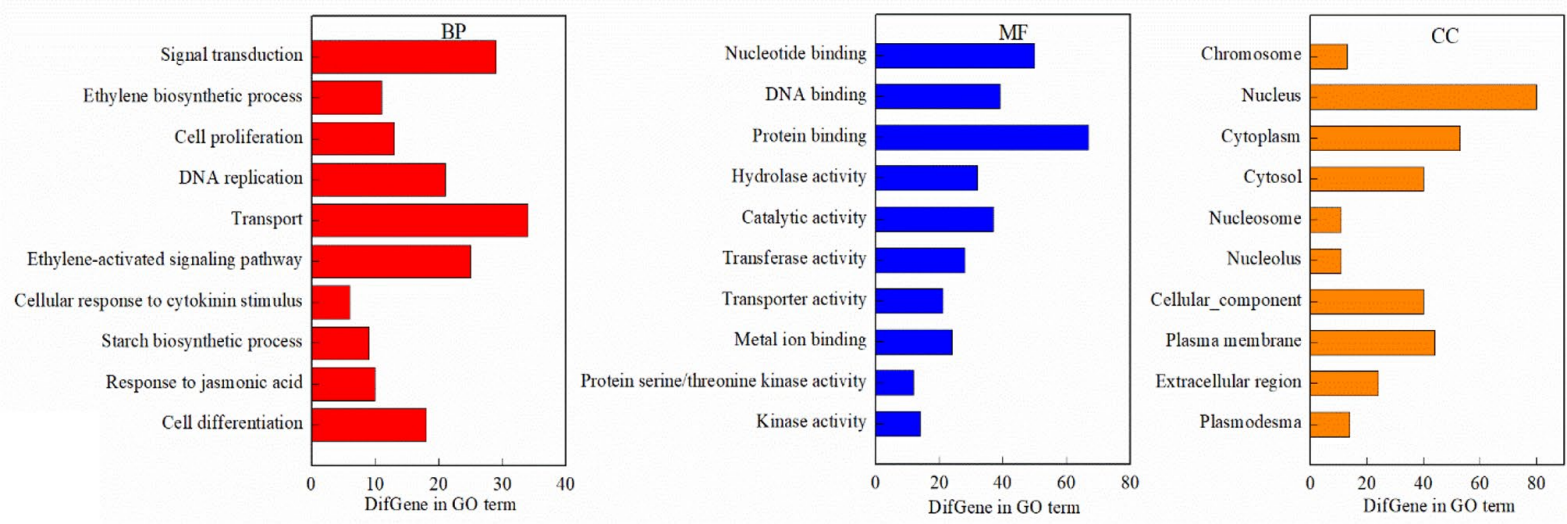
The Gene Ontology (GO) terms of the DEGs (FDR<0.05). BP, biological process; MF, molecular function; CC, cellular component.

GO analysis revealed that DEGs associated with the biological process (BP) term were largely associated with signal transduction, the ETH biosynthetic process, transport, the starch biosynthetic process, cell differentiation, and cell response to cytokinin (CTK) stimuli, while concerning the molecular function (MF) term, the DEGs were found to be associated with nucleotides; DNA and protein binding; and hydrolase, catalytic, transferase, transporter, and kinase activities (**Figure 9**). In addition, the DEGs associated with the cellular component (CC) term were largely associated with chromosomes, nuclei, the cytoplasm, CCs, the plasma membrane, and the extracellular region.

### Carbohydrate in Grain

**Figure 10:**
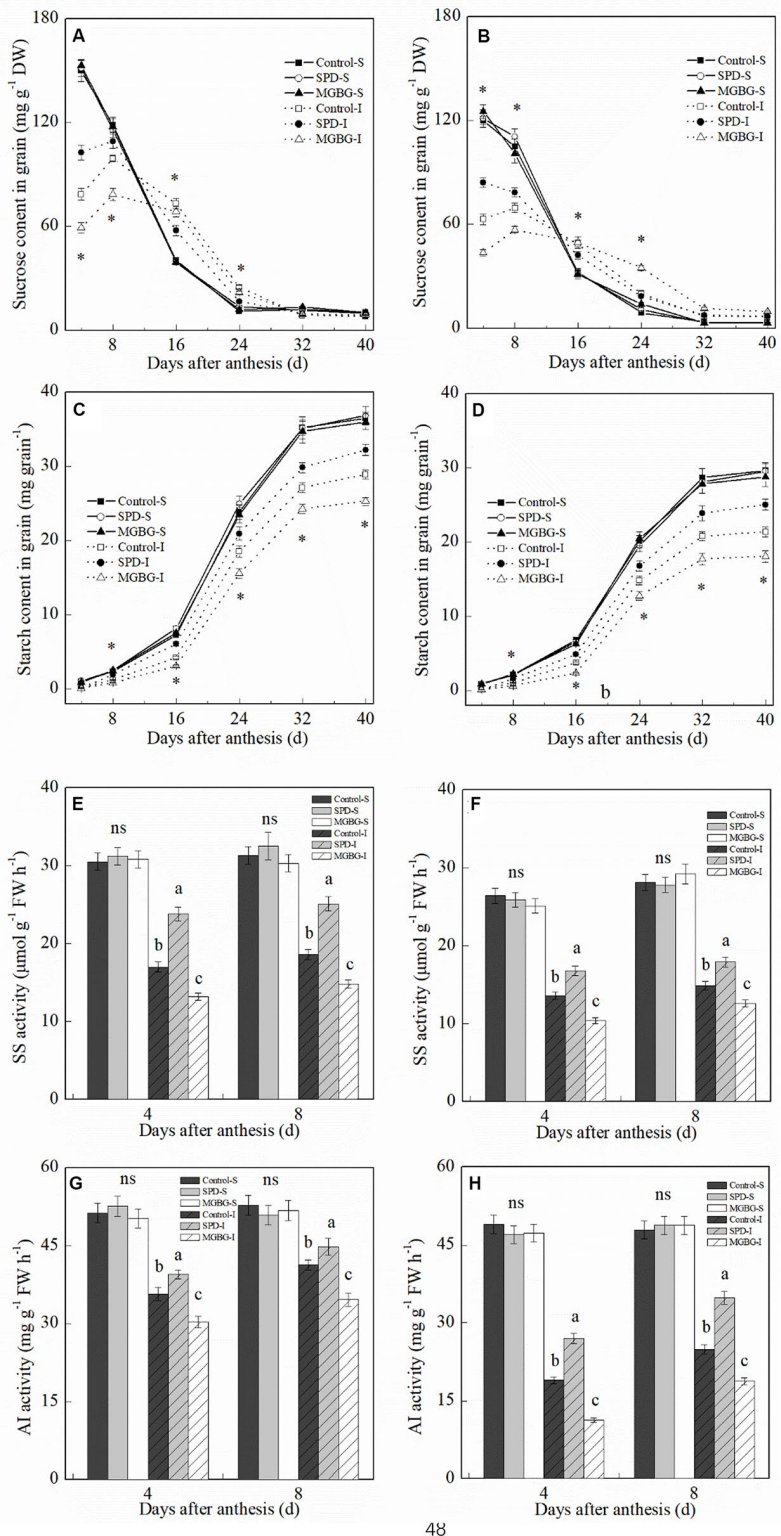
Effect of external PA on the sucrose and starch contents and sucrose synthase (SS) and acid invertase (AI) activities in grains of Shuangda 1 **(A**, **C**, **E**, and **G)** and Xinong 538 **(B**, **D**, **F**, and **H)**. SPD, MGBG, and control represent external applied Spd, MGBG, and water, respectively, to spikelets at anthesis stage. Vertical bars represent ± the standard error of the mean (n = 3, n represents the biological replicates). S, superior grain; I, inferior grain. E–H, Values within the same day and same grain type followed by different letters are significantly different (P 0.05). ns represents there being no significant difference among all of the treatments for the same grain type. * represents there being significant difference among the treatments for the same grain type (F value>F_0.05_).

During the grain-filling period, the sucrose content in the grain continually decreased, and the starch content in grain continually increased (**Figures 10A–D**). At 4 and 8 days post-anthesis, the sucrose content in the inferior grain was significantly lower than that in the superior grain. However, the sucrose content in the inferior grain was significantly greater than that in the superior grain at 16–24 days post-anthesis. In contrast to sucrose, the starch content in the inferior grain was significantly lower than that in the superior grain during the grain-filling period. Exogenous Spd and MGBG significantly altered the sucrose and starch contents in the inferior grain but had no significant effect on the sucrose and starch contents in the superior grain. Exogenous Spd significantly promoted an increase in sucrose content in the inferior grain at 4 and 8 days post-anthesis and an increase in starch content in the inferior grain at 16 and 40 days post-anthesis; exogenous MGBG had the opposite effect. In addition, at 4 and 8 days post-anthesis, exogenous Spd significantly promoted SS and AI activities in the inferior grain but had no significant effect on the SS and AI activities in the superior grain (**Figures 10E–H**); exogenous MGBG had the opposite effect.

### Hormones

**Figure 11:**
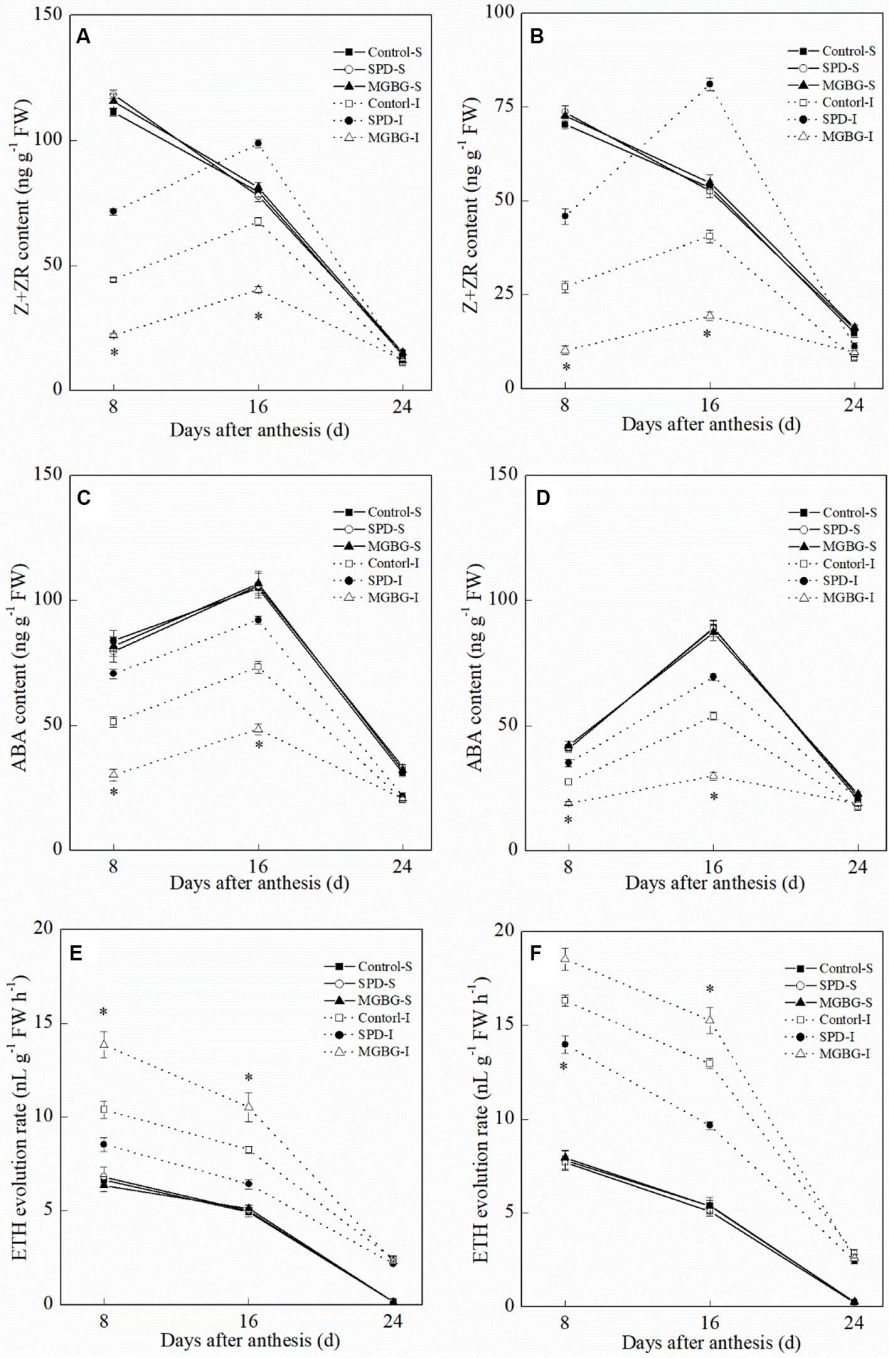
Effect of external PA on the hormonal changes in grains of Shuangda 1 **(A**, **C**, and **E)** and Xinong 538 **(B**, **D**, and **F)**. SPD, MGBG, and CK represent external applied Spd, MGBG, and water, respectively, to spikelets at anthesis stage. Vertical bars represent ± the standard error of the mean (n = 3, n represents the biological replicates). * represents there being significant difference among the treatments for the same grain type (F value>F_0.05_). S, superior grain; I, inferior grain.

Exogenous Spd significantly affected the Z+ZR, ABA, and ETH levels in the inferior grain but had no significant effect on those in the superior grain (**Figure 11**). Exogenous Spd significantly promoted an increase in Z+ZR and ABA levels in the inferior grain at 8 and 16 days post-anthesis. In contrast, the ETH evolution rate in the inferior grain in the SPD treatment was significantly lower than that in the CK treatment at 8 and 16 days post-anthesis; exogenous MGBG had the opposite effect.

## Discussion

How to improve the poor grain-filling ability of inferior grain in cereals such as rice and wheat is important for promoting wheat thousand grain weight and yield (Yang and Zhang, 2010). Previous studies have suggested that PAs are notably involved in the grain filling of rice and wheat (Wang et al., 2012; Liu et al., 2013) Yang et al. (2008) suggested that a high Spd level in grain is one of the reasons why superior grain had a higher grain-filling rate than did inferior grain. In addition, exogenous Spd notably released the inhibitory effect of drought on wheat grain filling (Yang et al., 2014;Liu et al., 2016). In the present study, the superior grain had a higher Spd level than did the inferior grain, and the Spd levels in grain of the high-grain-weight cultivars were notably higher than those of the low-grain-weight cultivars. In addition, correlation analysis revealed that the Spd level in the grain was positively and significantly correlated with the grain-filling rate and thousand grain weight. Exogenous Spd significantly increased grain filling. These results indicate that increased Spd levels in grain can notably promote increased grain filling in wheat. In contrast to the Spd content, the Put content was significantly greater in the inferior grain than in the superior grain, and the Put level in the grain was negatively and significantly correlated with the grain-filling rate and thousand grain weight. However, exogenous Put had no significant effect on the wheat grain filling, which means that Put may not be the key factor that regulates grain filling in wheat.

ADC, SAMDC, and SpdSy are key enzymes that regulate PA synthesis and are significantly involved in the regulation of grain filling in rice and wheat (Yang et al., 2008; Liu et al., 2016). ADC regulates Put synthesis, and SAMDC and SpdSy are involved in the pathway in which Spd is synthesized from Put (Chen et al., 2013). In the present study, the ADC activity in the grain did not significantly differ between the high-weight cultivars and the low-weight cultivars. However, the SAMDC and SpdSy activities in the grain of the high-grain-weight cultivars were significantly higher than those of the low-grain-weight cultivars, and the superior grain had higher SAMDC and SpdSy activities than did the inferior grain for the same cultivar. Correlation analysis suggested that SAMDC and SpdSy activities were positively and significantly correlated with Spd levels in the grain but were negatively and significantly correlated with Put levels (data not shown). However, ADC activity was not significantly correlated with PAs levels in the grain. This finding means that the synthesis of Put may not be significantly related to wheat thousand grain weight. In contrast, compared with the low-grain-weight cultivars and inferior grain, the high-grain-weight cultivars and superior grain had a more direct Put-to-Spd biosynthesis pathway. These results suggest that the direct synthetic pathway from Put to Spd within grain may be in favor of the grain filling and promoting the thousand grain weight in wheat.

The grain filling in wheat is governed by the grain-filling rate and the active grain-filling period. In the present study, compared with the superior grain, the significant low grain weight of inferior grain for these six cultivars seems due to the lower level of grain-filling rate (**Figure 1** and **Table 1**). In addition, the Put and Spd levels in the grain were not significantly correlated with the active grain-filling period. Exogenous Spd had no significant effect on the active grain-filling period, although it notably increased the wheat grain-filling rate and thousand grain weight. In addition, our results showed that exogenous Spd affected the grain-filling rate mainly during the early grain-filling period. The combination of the results in which exogenous Spd and MGBG significantly affected only the grain filling of inferior grain and not the grain filling of superior grain suggests that PAs regulated the grain-filling rate of the inferior grain mainly during the early grain-filling period, affecting the grain filling and thousand grain weight of wheat. Therefore, in the present study, we selected the inferior grain during the early grain-filling period (10 days post-anthesis) to research the regulatory mechanism of Spd in wheat grain filling.

Fu et al. (2011) suggested that sink strength is an important factor in regulating grain filling in rice. Low sink strength is one of the main reasons for the poor grain filling of inferior rice and wheat grain, and increased sink strength notably improves the poor grain filling of inferior cereal grain (Kato, 2004). With respect to the synthesis of starch from sucrose in grain, endogenous hormone levels are important indices of sink strength in cereals (Ishimaru et al., 2005; Wang et al., 2008). In the present study, exogenous Spd significantly promoted an increase in sucrose content in the inferior grain during the early grain-filling period, and MGBG had the opposite effect. In addition, the SS and AI activities in the inferior grain in the Spd treatment were significantly higher than those in the control treatment, and MGBG notably reduced the activities of both enzymes. The previous study suggested that the high SS activity in grain promotes the synthetic from sucrose to starch, and this effect was in favor to the sucrose transport from stem to grain (Jiang et al., 2003; Yang et al., 2004). Beside this, the AI was related to the sucrose unloading in grain, and the nitrogen application notably promoted the AI activity in wheat grain and increased the sugar content in grains (Zhang et al., 2014). SS and AI activities notably promote increased sink activity, and the activity of these two enzymes is positively and significantly correlated with the grain-filling rate in rice and wheat (Verma et al., 2018). These results indicate that Spd notably promoted the unloading of sucrose, which was then transported from source tissue, in the sink tissue of inferior grain. Zhang et al. (2009) suggested that one of the reasons for the poor grain filling of inferior rice grain is the “energy barrier” of inferior grain, which has difficulty receiving sucrose transported from stem source tissue. On the basis of these findings, we suggested that the promotive effect of Spd on the unloading of sucrose in inferior grain may significantly increase the reception ability of inferior grain for sucrose, thus promoting the transport of sucrose from the stem to inferior grain.

Previous studies have suggested that multiple plant hormones are involved in the regulation of grain filling in cereals. CTKs represent an important factor that is significantly related to grain filling in cereals. CTK levels in roots were positively correlated with photosynthesis in the flag leaves of rice during the grain-filling period (Yang et al., 2002). Moreover, grain-filling patterns were significantly related to Z+ZR contents in both the grain and the roots during the early and middle grain-filling periods (Yang et al., 2000). The results of our previous study suggested that drought stress significantly reduced Z+ZR contents in grain and inhibited the grain filling of wheat (Liu et al., 2013b). In the present study, exogenous Spd significantly promoted an increase in Z+ZR contents in the inferior grain at 8 and 16 days post-anthesis; exogenous MGBG had the opposite effect. Previous studies suggested that the effects of PA on rice grain filling were notably related to Z+ZR contents in the grain (Yang et al., 2008). In addition, the results of our previous study suggested that Spd notably relieves the inhibition effect of drought on grain filling of wheat, and this may relate to the significant increasing of the Z+ZR concentrations in the grains. However, the external Put had no significant effect on the Z+ZR concentrations in grains, and it cannot relieve the inhibition effect of drought on grain filling of wheat (Liu et al., 2016). These findings mean that the promotive effect of Spd on the filling of inferior wheat grain is notably related to the Z+ZR content. GO analysis in the present study revealed that exogenous Spd significantly affected the BPs involving cellular responses to CTK stimulus and cell differentiation (**Figure 6D**). The number and division rate of endosperm cells determine the sink size in cereals such as wheat and rice (Chen et al., 2013). Zhang et al. (2010) suggested that CTKs significantly increase endosperm cell division and promote the sink strength of rice grain. In addition, CTK levels notably increased during the early development of the seeds of both pea and bean (Michael and Seiler-Kelbitsch 1972; Saha et al., 1986; Morris et al., 1993; Dietrich et al., 1995). Beside this, CTK was notably related to the sucrose metabolism. Yang et al. (2002) suggested that ABA and CTK are involved in controlling plant senescence and enhanced carbon remobilization when wheat is subjected to water stress. Lee and Huang (2013) suggested that CTK affects sucrose metabolism conducing to *de novo* shoot organogenesis in rice callus. Wang et al. (2016) suggested that lovastatin, a CTK inhibitor, inhibited the invertase activity and metabolism and transport of glucose, fructose, and sucrose of *tasg1*, a wheat stay-green mutant; however, the activity of invertase was partially recovered in *tasg1* when treated with 6-benzylaminopurine, and this means that CTK might regulate the stay-green phenotype of *tasg1* by regulating the invertase activity involved in sucrose remobilization. Our previous study suggested that the Z+ZR contents in grain were significantly and positively correlated with the SS activities in wheat grain and that a high Z+ZR content in grain promoted the synthesis of starch, which promoted the grain filling of wheat (Liang et al., 2017). These mean that CTK significantly regulated the sucrose metabolism and was involved in the synthesis from sucrose to starch during wheat grain filling. These results suggested that Spd increased the Z+ZR contents in the inferior grain of wheat and promoted endosperm cell division, thereby increasing the sink size of the inferior grain. These phenomena caused the inferior grain to accommodate increased amounts of carbohydrates transported from the stem. In addition, exogenous Spd also promoted SS and AI activities and sucrose unloading in the inferior grain, which means that Spd may affect CTK levels in the grain to promote both increased sink size and sink strength in the inferior grain and the transport of sucrose from the stem to that grain. These phenomena may constitute the main reason why Spd promoted increased filling of the inferior wheat grain.

In addition to CTKs, ETH and ABA are also involved in the regulation of grain filling of cereals. Yang et al. (2006) suggested that high ratio of ABA/ETH under soil drought stress notably promoted the grain-filling rate of wheat. Lv et al. (2017) suggested that foliar applications of potassium significantly promoted the ABA concentration and reduced ETH evolution rates in inferior grain and increased the grain-filling rate of inferior grain in wheat. PAs and ETH share the same synthetic precursor, and their metabolism is affected by each other (Bisbis et al., 2000). Wang et al. (2012) suggested that the interaction between PAs and ETH notably regulated rice grain filling. Liu et al. (2013) suggested that the promoting effect of external Spd on grain filling of wheat was significantly related to the increasing of ABA concentration in grains. In the present study, exogenous Spd significantly reduced the ETH evolution rate and promoted the ABA concentration in the inferior grain, whereas MGBG had the opposite effect. These results are similar to those of previous studies (Chen et al., 2013;Liu et al., 2013). In addition, previous studies suggested that ETH inhibits wheat endosperm cell division (Yang et al., 2017). The ABA increased the sink strength of the inferior grain and promoted the carbohydrate transport to inferior grain of rice (Yang and Zhang, 2010). These results mean that Spd maybe inhibited ETH synthesis and promoted the ABA concentration to promote the endosperm cell division and sink strength of wheat grain.

## Conclusion

PAs significantly affected wheat grain filling and thousand grain weight. The direct synthetic pathway of Spd from Put in the grain was a key factor in promoting increased grain filling and thousand grain weight in wheat. Spd regulates the grain-filling rate of inferior grain mainly during the early grain-filling period, affecting wheat grain filling and thousand grain weight. The promotive effect of Spd on the grain filling of the inferior grain of wheat was notably related to carbohydrate transport from the stem to that grain. Spd significantly increased the Z+ZR contents but reduced the ETH evolution rate in the inferior grain. In addition, Spd significantly increased the SS and AI activities in the inferior grain. These effects of Spd led to increased sucrose content in the inferior grain. Together, these findings may be some of the main reasons why Spd significantly promoted the increased filling and weight of the inferior wheat grain.

## Data Availability Statement

The RNA-seq data were deposited in the Sequence Read Archive of NCBI; the accession number is SRP217735.

## Author Contributions

Conceived and designed the experiments: YaL. Performed the experiments: JL, BW. Analyzed the data: JH, BW. Wrote the paper: YaL, JL, YuL.

## Conflict of Interest

The authors declare that the research was conducted in the absence of any commercial or financial relationships that could be construed as a potential conflict of interest.

## References

[B1] Ashburner M. Ball C. A. Blake J. A. Botstein D. Butler H. Cherry J. M. (2000). Gene ontology: tool for the unification of biology. The Gene Ontology Consortium. Nat. Genet. 25. 10.1038/75556 10802651 PMC3037419

[B2] Beltrano J. Carbone A. Montaldi E. R. Guiamet J. J. (1994). Ethylene as promoter of wheat grain maturation and ear senescence. Plant Growth Regul. 15, 107–112. 10.1007/BF00024098

[B3] Bisbis B. Kevers C. Dommes J. Gaspar T. (2000). Interactions between polyamine and ethylene metabolisms in a hormone autonomous sugarbeet callus. J. Plant Physiol. 157, 24–30. 10.1016/S0176-1617(00)80131-5

[B4] Cai T. Xu H. C. Peng D. L. Yin Y. P. Yang W. B. Ni Y. L. (2014). Exogenous hormonal application improves grain yield of wheat by optimizing tiller productivity. Field Crops Res. 155, 172–183. 10.1016/j.fcr.2013.09.008

[B5] Chen T. T. Xu Y. J. Wang J. C. Wang Z. Q. Yang J. C. Zhang J. H. (2013). Polyamines and ethylene interact in rice grains in response to soil drying during grain filling. J. Exp. Bot. 64, 2523–2536. 10.1093/jxb/ert115 23606413

[B6] Dietrich J. T. Kaminek M. Blevins D. G. Reinbott T. M. Morris R. O. (1995). Changes in cytokinins and cytokinin oxidase activity in developing maize kernels and the effects of exogenous cytokinin on kernel development. Plant Physiol. Bioch. 33, 327–336.

[B7] Dwivedi S. K. Basu S. Kumar S. Kumar G. Prakash V. Kumar S. (2017). Heat stress induced impairment of starch mobilisation regulates pollen viability and grain yield in wheat: Study in Eastern Indo-Gangetic Plains. Field Crops Res. 206, 106–114. 10.1016/j.fcr.2017.03.006

[B8] Feng-Peng L. Min-Young Y. Gang L. Won-Hee R. Jae-Wan P. Soon-Jae K. (2014). Transcriptome analysis of grain-filling caryopses reveals the potential formation mechanism of the rice sugary mutant. Gene 546, 318–326. 10.1016/j.gene.2014.05.059 24875416

[B9] Fu J. Huang Z. Wang Z. Yang J. Zhang J. (2011). Pre-anthesis non-structural carbohydrate reserve in the stem enhances the sink strength of inferior spikelets during grain filling of rice. Field Crops Res. 123, 170–182. 10.1016/j.fcr.2011.05.015

[B10] Hao Z. Tan G. Yang L. Yang J. Zhang J. Zhao B. (2009). Hormones in the grains and roots in relation to post-anthesis development of inferior and superior spikelets in japonica/indica hybrid rice. Plant Physiol. Biochem. 47, 195–204. 10.1016/j.plaphy.2008.11.012 19117763

[B11] Ishimaru T. Hirose T. Matsuda T. Goto A. Takahashi K. Sasaki H. (2005). Expression patterns of genes encoding carbohydrate-metabolizing enzymes and their relationship to grain-filling in rice (Oryza sativa L.): comparison of caryopses located at different positions in a panicle. Plant Cell Physiol. 46, 620–628. 10.1093/pcp/pci066 15701658

[B12] Jeong K. Baten A. Waters D. L. Pantoja O. Julia C. C. Wissuwa M. (2017). Phosphorus remobilisation from rice flag leaves during grain filling: an RNA-seq study. Plant Biotechnol. J. 15, 15–26. 10.1111/pbi.12586 27228336 PMC5253468

[B13] Jiang D. Cao W. X. Dai T. B. Jing Q. (2003). Activities of key enzymes for starch synthesis in relation to growth of superior and inferior grains on winter wheat (Triticum aestivum L.) spike. Plant Growth Regul. 41 (3), 247–257. 10.1023/B:GROW.0000007500.90240.7d

[B14] Kasukabe Y. He L. Nada K. Misawa S. Ihara I. Tachibana S. (2004). Overexpression of spermidine synthase enhances tolerance to multiple environmental stress and up-regulates the expression of various stress-regulated genes in transgenic Arabidopsis thaliana. Plant Cell Physiol. 45, 712–722. 10.1093/pcp/pch083 15215506

[B15] Kato T. (2004). Effect of spikelet removal on the grain filling of Akenohoshi, a rice cultivar with numerous spikelets in a panicle. J. Agr. Sci. 142, 177–181. 10.1017/S0021859604004265

[B16] Kato T. Shinmura D. Taniguchi A. (2007). Activities of enzymes for sucrose-starch conversion in developing endosperm of rice and their association with grain-filling in extra-heavy panicle types. Plant Prod. Sci. 10, 442–450. 10.1626/pps.10.442

[B17] Kelbert A. J. Spaner D. Briggs K. G. King J. R. (2004). Screening for lodging resistance in spring wheat breeding programmes. Plant Breed. 123, 349–354. 10.1111/j.1439-0523.2004.00976.x

[B18] Kim J. Shon J. Lee C. K. Yang W. Yoon Y. Yang W. H. (2011). Relationship between grain filling duration and leaf senescence of temperate rice under high temperature. Field Crops Res. 122, 207–213. 10.1016/j.fcr.2011.03.014

[B19] Kusano T. Yamaguchi K. Berberich T. Takahashi Y. (2007). Advances in polyamine research in J. Plant Res. 120, 345–350. 10.1007/s10265-007-0074-3 17351711

[B20] Langmead B. Trapnell C. Pop M. Salzberg S. L. (2009). Ultrafast and memory-efficient alignment of short DNA sequencesto the human genome. Genome Biol. 10 (3), R25. 10.1186/gb-2009-10-3-r25 19261174 PMC2690996

[B21] Lee S. T. Huang W. L. (2013). Cytokinin, auxin, and abscisic acid affects sucrose metabolism conduce to de novo shoot organogenesis in rice (Oryza sativa L.) callus. Bot Stud. 54, 5. 10.1186/1999-3110-54-5 28510848 PMC5383921

[B22] Lee T. M. Lur H. S. Chu C. (1997). Role of abscisic acid in chilling tolerance of rice (Oryza sativa L) seedlings.2. Modulation Free polyamine levels. Plant Sci. 126, 1–10. 10.1016/S0168-9452(97)00076-9

[B23] Liang W. Zhang Z. Wen X. Liao Y. Liu Y. (2017). Effect of non-structural carbohydrate accumulation in the stem pre-anthesis on grain filling of wheat inferior grain. Field Crops Res. 211, 66–76. 10.1016/j.fcr.2017.06.016

[B24] Liu Y. Wang Q. S. Ding Y. F. Li G. H. Xu J. X. Wang S. H. (2011). Effects of external ABA, GA3 and NAA on the tiller bud outgrowth of rice is related to changes in endogenous hormones. Plant Growth Regu. 65, 247–254. 10.1007/s10725-011-9594-x

[B25] Liu J. Ji X. J. Liu Y. L. (2002). High performance liquid chromatography method for measuring polymine content in plant tissue. Plant Physiol. Commun. 38, 596–599.

[B26] Liu Y. Gu D. Wu W. Wen X. Liao Y. (2013a). The relationship between polyamines and hormones in the regulation of wheat grain filling. PloS One 8 (10), e78196. 10.1371/journal.pone.0078196 24205154 PMC3812141

[B27] Liu Y. Liang H. Y. Lv X. K. Liu D. D. Wen X. X. Liao Y. C. (2016). Effect of polyamines on the grain filling of wheat under drought stress. Plant Physiol. Bioch. 100, 113–129. 10.1016/j.plaphy.2016.01.003 26812255

[B28] Liu Y. Sui Y. W. Gu D. D. Wen X. X. Chen Y. Li C. (2013b). Effects of conservation tillage on grain filling and hormonal changes in wheat under simulated rainfall conditions. Field Crops Res. 144, 43–51. 10.1016/j.fcr.2013.01.009

[B29] Li F. P. Yoon M. Y. Li G. Ra W. H. Park J. W. Kwon S. J. (2014). Transcriptome analysis of grain-filling caryopses reveals the potential formation mechanism of the rice sugary mutant. Gene. 546, 318–326. 10.1016/j.gene.2014.05.059 24875416

[B30] Lv X. Li T. Wen X. Liao Y. Liu Y. (2017). Effect of potassium foliage application post-anthesis on grain filling of wheat under drought stress. Field Crops Res. 206, 95–105. 10.1016/j.fcr.2017.02.015

[B31] Michael G. Seiler-Kelbitsch H. (1972). Cytokinin content and kernel size of barley grains as affected by environmental and genetic factors. Crop Sci. 12, 162–165. 10.2135/cropsci1972.0011183X001200020002x

[B32] Morris R. O. Blevins D. G. Dietrich J. T. Durley R. C. Gelvin S. B. Gray J. (1993). Cytokinins in plant-pathogenic bacteria and developing cereal-grains. Aust. J. Plant Physiol. 20, 621–637. 10.1071/PP9930621

[B33] Murty P. S. S. Murty K. S. (1982). Spikelet sterility in relation to nitrogen and carbohydrate contents in rice. Indian J. Plant Physiol. 25, 40–48.

[B34] Oufir M. Legay S. Nicot N. Van Moer K. Hoffmann L. Renaut J. (2008). Gene expression in potato during cold exposure: Changes in carbohydrate and polyamine metabolisms. Plant Sci. 175, 839–852. 10.1016/j.plantsci.2008.08.010

[B35] Peng T. Du Y. Zhang J. Li J. Liu Y. Zhao Y. (2013). Genome-wide analysis of 24-nt siRNAs dynamic variations during rice superior and inferior grain filling. PloS One 8, e61029. 10.1371/journal.pone.0061029 23593380 PMC3625182

[B36] Peng T. Lv Q. Zhang J. Li J. Du Y. Zhao Q. (2011). Differential expression of the microRNAs in superior and inferior spikelets in rice (Oryza sativa). J. Exp. Bot. 62, 4943–4954. 10.1093/jxb/err205 21791435

[B37] Pertea M. Kim D. Pertea G. M. Leek J. T. Salzberg S. L. (2016). Transcript-level expression analysis of RNA-seq experiments with HISAT, StringTie and Ballgown. Nat. Protoc. 11, 1650. 10.1038/nprot.2016.095 27560171 PMC5032908

[B38] Richards F. J. (1959). A ﬂexible growth function for empirical use. J. Exp. Bot. 10, 290–300. 10.1093/jxb/10.2.290

[B39] Robert N. (2002). Comparison of stability statistics for yield and quality traits in bread wheat. Euphytica 128, 333–341. 10.1023/A:1021296919225

[B40] Saha S. Nagar P. K. Sircar P. K. (1986). Cytokinin concentration gradient in the developing grains and upper leaves of rice (Oryza sativa) during grain filling. Can. J. Bot. 64, 2068–2072. 10.1139/b86-271

[B41] Sicher R. C. Bunce J. A. (1998). Evidence that Premature Senescence Affects Photosynthetic Decline of Wheat Flag Leaves during Growth in Elevated Carbon Dioxide. Int. J. Plant Sci. 159, 798–804. 10.1086/297599

[B42] Sikder H. P. Gupta D. K. D. (1976). Physiology of grain in rice. Indian Agric. 20, 133–141.

[B43] Takahashi T. Nagao K. Itagaki H. Takaku T. Tsuchihashi N. Nakaseko K. (1996). Grain filling mechanisms in spring wheat.6. Cultivar variation in nitrogen metabolism and changes in assimilate shortages. Jap. J. Crop Sci. 65, 289–295. 10.1626/jcs.65.289

[B44] Trapnell C. Pachter L. Salzberg S. L. (2009). TopHat: discoveringsplice junctions with RNA-Seq. Bioinformatics 25 (9), 1105–1111. 10.1093/bioinformatics/btp120 19289445 PMC2672628

[B45] Verma E. Sharma B. Singal H. R. Munjal R. (2018). Purification of sucrose synthase from thermotolerant wheat grains and its characterization. J. Environ. Biol. 39, 459–466. 10.22438/jeb/39/4/MRN-503

[B46] Wang E. T. Wang J. J. Zhu X. D. Hao W. Wang L. Y. Li Q. (2008). Control of rice grain-filling and yield by a gene with a potential signature of domestication. Nat. Genet. 40, 1370–1374. 10.1038/ng.220 18820698

[B47] Wang G. Q. Hao S. S. Gao B. Chen M. X. Liu Y. G. Yang J. C. (2017). Regulation of gene expression in the remobilization of carbon reserves in rice stems during grain filling. Plant Cell Physiol. 58, 1391–1404. 10.1093/pcp/pcx072 28575477

[B48] Wang W. Hao Q. Tian F. Li Q Wang W. (2016). Cytokinin-regulated sucrose metabolism in stay-green wheat phenotype. PloS One, 11, e0161351. 10.1371/journal.pone.0161351 27580166 PMC5007033

[B49] Wang X. K. Huang J. L., (2000). Principles and Techniques of Plant Physiological Biochemical Experiment. Beijing: Higher Education Press 2000.

[B50] Wang Z. Xu Y. Wang J. Yang J. (2012). Polyamine and ethylene interactions in grain filling of superior and inferior spikelets of rice. Plant Growth Reg. 66, 215–228. 10.1007/s10725-011-9644-4

[B51] Wilkinson S. Kudoyarova G. R. Veselov D. S. Arkhipova T. N. Davies W. J. (2012). Plant hormone interactions: innovative targets for crop breeding and management. J. Exp. Bot. 63, 3499–3509. 10.1093/jxb/ers148 22641615

[B52] Yang J. C. Cao Y. Y. Zhang H. Liu L. J. Zhang J. H. (2008). Involvement of polyamines in the post-anthesis development of inferior and superior spikelets in rice. Planta 228, 137–149. 10.1007/s00425-008-0725-1 18340459

[B53] Yang J. Peng S. Visperas R. M. Sanico A. L. Zhu Q. Gu S. (2000). Grain filling pattern and cytokinin content in the grains and roots of rice plants. Plant Growth Reg. 30, 261–270. 10.1023/A:1006356125418

[B54] Yang J. C. Zhang J. H. (2010). Grain filling problem in 'super' rice. J. Exp. Bot. 61, 1–5. 10.1093/jxb/erp348 19959608

[B55] Yang J. Zhang J. Wang Z. Zhu Q. Liu L. (2002). Abscisic acid and cytokinins in the root exudates and leaves and their relationship to senescence and remobilization of carbon reserves in rice subjected to water stress during grain filling. Planta 215, 645–652. 10.1007/s00425-002-0789-2 12172848

[B56] Yang J. C. Zhang J. H. Wang Z. Q. Xu G. W. Zhu Q. S. (2004). Activities of key enzymes in sucrose-to-starch conversion in wheat grains subjectedto water deficit during grain filling. Plant Physiol. 135, 1621–1629. 10.1104/pp.104.041038 15235118 PMC519076

[B57] Yang J. C. Zhang J. H. (2006). Grain filling of cereals under soil drying. New Phytol. 169, 223–236. 10.1111/j.1469-8137.2005.01597.x 16411926

[B58] Yang J. C. Zhang J. H. Liu K. Wang Z. Q. Liu L. J. (2006). Abscisic acid and ethylene interact in wheat grains in response to soil drying during grain filling. New Phytol. 171 (2), 293–303. 10.1111/j.1469-8137.2006.01753.x 16866937

[B59] Yang W. Li Y. Yin Y. Qin Z. Zheng M. Chen J. (2017). Involvement of ethylene and polyamines biosynthesis and abdominal phloem tissues characters of wheat caryopsis during grain filling under stress conditions. Sci. Rep. 7, 46020. 10.1038/srep46020 28383077 PMC5382545

[B60] Yang W. B. Yin Y. P. Li Y. Cai T. Ni Y. L. Peng D. L. (2014). Interactions between polyamines and ethylene during grain filling in wheat grown under water deficit conditions. Plant Growth Regul., 72, 189–201. 10.1007/s10725-013-9851-2

[B61] Yoshihisa K. Lixiong H. Kazuyoshi N. Shuhei M. Izumi I. Shoji T. (2004). Overexpression of spermidine synthase enhances tolerance to multiple environmental stresses and up-regulates the expression of various stress-regulated genes in transgenic Arabidopsis thaliana. Plant Cell Physiol. 45, 712. 10.1093/pcp/pch083 15215506

[B62] Yu Z. W. (2011). “Technical regulations for high-yield wheat cultivation in China,” in Ministry of Agriculture of the People's Republic of China. China Agriculture Press.

[B63] Zhang H. Chen T. Wang Z. Yang J. Zhang J. (2010). Involvement of cytokinins in the grain filling of rice under alternate wetting and drying irrigation. J. Exp. Bot. 61, 3719–3733. 10.1093/jxb/erq198 20584789

[B64] Zhang H. Tan G. L. Yang L. N. Yang J. C. Zhang J. H. Zhao B. H. (2009). Hormones in the grains and roots in relation to post-anthesis development of inferior and superior spikelets in japonica/indica hybrid rice. Plant Physiol. Bioch. 47, 195-204. 10.1016/j.plaphy.2008.11.012 19117763

[B65] Zhang Y. F. Ma D. Y. Wang C. Y. Zhu Y. J. Xie Y. X. Guo T. C. (2014). Effects of nitrogen application on endosperm cell proliferating, activity of cell wall invertase and sugar content in grains of wheat cultivars with different grain size. J. Triticeae Crops 34 (1), 96–101. (in Chinese with English abstract).

[B66] Zhu Q. S. Cao X. Luo Y. (1988). Growth analysis in the process of grain ﬁlling in rice. Acta Agron. Sin. 14, 182–192.

