# Supplementary material for: Spermidine Increases the Sucrose Content in Inferior Grain of Wheat and Thereby Promotes Its Grain Filling
Source: Front Plant Sci. 2019 Nov 21;10:1309. doi: 10.3389/fpls.2019.01309 (PMC6881305; doi:10.3389/fpls.2019.01309)
Supplement: Supplementary file 1 [file Table_1.docx]

**Supplemental Table 1 Grain yield and yield** **components of different wheat cultivars**

| Year | Cultivars | No. of panicles | Spikelets per panicle | Thousand grain weight | Grain yield |
| --- | --- | --- | --- | --- | --- |
|  |  | (× 10^4^ hm^-2^) |  | (g) | (t hm^-2^) |
| 2014-2015 | Shuagda 1 | 370.02c | 38.95a | 57.84a | 7.55ab |
|  | Fugao 1 | 525.03b | 37.37a | 45.95b | 7.91a |
|  | Zhoumai 22 | 510.51b | 37.58a | 47.83b | 7.96a |
|  | Xiaoyan 6 | 615.34a | 33.16b | 35.85c | 7.13b |
|  | Xiaoyan 22 | 630.81a | 36.18ab | 36.34c | 7.44ab |
|  | Xinong 538 | 674.86a | 34.56b | 37.57c | 7.70a |
| 2015-2016 | Shuagda 1 | 327.62c | 40.31a | 50.16a | 6.95ab |
|  | Fugao 1 | 493.85b | 39.35a | 41.24b | 7.84a |
|  | Zhoumai 22 | 451.46b | 38.46a | 42.11b | 7.38a |
|  | Xiaoyan 6 | 584.11a | 31.67b | 31.57c | 6.02b |
|  | Xiaoyan 22 | 601.95a | 33.95b | 34.61c | 6.81b |
|  | Xinong 538 | 612.79a | 32.57b | 30.15c | 6.25b |
|  |  |  |  |  |  |

Values within a column and for the same year followed by different letters are significantly different (P<0.05).
