# Supplementary material for: Spermidine Increases the Sucrose Content in Inferior Grain of Wheat and Thereby Promotes Its Grain Filling
Source: Front Plant Sci. 2019 Nov 21;10:1309. doi: 10.3389/fpls.2019.01309 (PMC6881305; doi:10.3389/fpls.2019.01309)
Supplement: Supplementary file 2 [file Table_2.docx]

**Supplemental Table 2 Effect of external PA on endogenous Spd and Put concentration in grains**

| Cultivar | Spikelet categories | Treatment | Spd (ng g^-1^ FW) | | Put (ng g^-1^ FW) | |
| --- | --- | --- | --- | --- | --- | --- |
|  |  |  | 12 DPA | 24 DPA | 12 DPA | 24 DPA |
| Shuangda 1 | S | Control | 39.21a | 17.42a | 20.27b | 9.70b |
|  |  | SPD | 40.45a | 19.14a | 18.51b | 11.46b |
|  |  | PUT | 41.02a | 18.88a | 19.97b | 10.22b |
|  |  | MGBG | 27.59b | 10.45b | 29.09a | 15.87a |
|  | I | Control | 23.22b | 11.33b | 14.10b | 6.17b |
|  |  | SPD | 34.36a | 17.57a | 13.22b | 5.45b |
|  |  | PUT | 24.68b | 12.05b | 13.82b | 5.75b |
|  |  | MGBG | 13.07c | 7.55c | 21.16a | 9.93a |
| Xinong 538 | S | Control | 15.90a | 10.89a | 43.31b | 13.22b |
|  |  | SPD | 17.59a | 11.91a | 41.43b | 12.34b |
|  |  | PUT | 17.42a | 10.59a | 40.82b | 14.68b |
|  |  | MGBG | 10.16b | 6.85b | 56.99a | 27.33a |
|  | I | Control | 8.71b | 4.52b | 62.59b | 21.16b |
|  |  | SPD | 18.88a | 8.28a | 60.82b | 22.92b |
|  |  | PUT | 10.62b | 4.79b | 61.85b | 22.38b |
|  |  | MGBG | 5.11c | 2.46c | 86.38a | 37.02a |

Values within a column and for the same cultivar and same grain type followed by different letters are significantly different (P<0.05). S: superior grain; I: inferior grain. DPA: days post anthesis. SPD, MGBG and Control represent external applied Spd, MGBG and water, respectively, to spikelets at anthesis stage.
