# Supplementary material for: Spermidine Increases the Sucrose Content in Inferior Grain of Wheat and Thereby Promotes Its Grain Filling
Source: Front Plant Sci. 2019 Nov 21;10:1309. doi: 10.3389/fpls.2019.01309 (PMC6881305; doi:10.3389/fpls.2019.01309)
Supplement: Supplementary file 3 [file Table_3.docx]

**Supplemental Table 3 Effect of external PA on grain number per spikes and thousand grain weight of wheat**

| Year | Cultivars | Treatment | Grain number per spikes | Thousand grain weight |
| --- | --- | --- | --- | --- |
|  |  |  |  | (g) |
| 2014-2015 | Shuagda 1 | Control | 39.02a | 57.15b |
|  |  | SPD | 38.97a | 59.34a |
|  |  | PUT | 39.14a | 56.57bc |
|  |  | MGBG | 38.56a | 55.23c |
|  | Xinong 538 | Control | 34.28a | 37.96b |
|  |  | SPD | 34.16a | 40.58a |
|  |  | PUT | 34.33a | 37.24b |
|  |  | MGBG | 34.45a | 35.03c |
| 2015-2016 | Shuagda 1 | Control | 39.54a | 50.87b |
|  |  | SPD | 39.16a | 53.06a |
|  |  | PUT | 38.98a | 50.34b |
|  |  | MGBG | 39.07a | 47.96c |
|  | Xinong 538 | Control | 32.86a | 30.94b |
|  |  | SPD | 32.77a | 33.85a |
|  |  | PUT | 33.18a | 30.22b |
|  |  | MGBG | 32.61a | 27.69c |

Values within a column and for the same year and same cultivar followed by different letters are significantly different (P<0.05). SPD, MGBG, PUT and Control represent external applied Spd, MGBG, Put and water, respectively, to spikelets at anthesis stage.
